# Sensory Stimulation and Music Therapy Programs for Treating Disorders of Consciousness

**DOI:** 10.3389/fpsyg.2016.00297

**Published:** 2016-03-07

**Authors:** Caroline Schnakers, Wendy L. Magee, Brian Harris

**Affiliations:** ^1^Department of Neurosurgery, University of California, Los AngelesLos Angeles, CA, USA; ^2^Music Therapy Program, Boyer College of Music and Dance, Temple UniversityPhiladelphia, PA, USA; ^3^Department of Physical Medicine and Rehabilitation, Spaulding Rehabilitation HospitalBoston, MA, USA

**Keywords:** brain injury, consciousness, sensory stimulation program, music therapy

## Introduction

Until now, no treatment has shown its efficacy in patients with severe brain injury, with the exception of one pharmacological agent (i.e., Amantadine; Giacino et al., 2012). Recovery of consciousness is therefore one of the biggest challenge facing clinicians (Whyte, 2014). For years, sensory stimulation programs have been the most frequently applied treatment during patients' neurorehabilitation (Tolle and Reimer, 2003). These programs are based on the idea that an enriched environment benefits brain plasticity and improves recovery of injured brains.

Theories of brain plasticity, which suggest that an adult injured brain has the capacity to reorganize itself to compensate for affected regions, have broadly been accepted for several years (Hummel and Cohen, 2005). The most famous case illustrating this phenomenon is the case of Terry Wallis (Voss et al., 2006). This patient remained in a minimally conscious state for 19 years after a traumatic brain injury and yet recovered functional verbal and motor activities. A study of this case revealed a neural change, mainly involving the precuneus which is related to consciousness, suggesting that this spectacular recovery could be explained by brain plasticity. These results stress the importance of developing therapeutics that intensify brain plasticity in severely brain-injured adults to reach full recovery of consciousness.

Providing sensory stimulation may potentially stimulate affected neural networks, accelerate brain plasticity, and avoid a sensory deprivation that could slow down the patient's recovery. The efficacy of such intervention is, however, still currently debated. Recently, music therapy has been presented as another potential way to stimulate those patients and may constitute a promising alternative to sensory stimulation programs (Magee and O'Kelly, 2015).

## Sensory stimulation: theoretical principles

Rosenzweig and colleagues introduced “environmental enrichment” in the field of animal research four decades ago to investigate the influence of environment on brain and behavior, and showed that the morphology and physiology of the brain can be altered by modifying the quality and intensity of environmental stimulation (Rosenzweig, 1966). An enriched environment is an environment with enhanced novel and complex stimulation relative to a standard environment, providing the animals with optimal conditions for enhanced exploration, cognitive activity and physical exercise (Rosenzweig et al., 1978). It has been associated with an increase in cortical thickness and weight (Rosenzweig et al., 1964; Beaulieu and Colonnier, 1987), size of the cell soma and nucleus, dendritic arborisation, length of dendritic spines (Holloway, 1966; Greenough et al., 1973; Kozorovitskiy et al., 2005) and synaptic size and number (Diamond et al., 1964; Mollgaard et al., 1971; Turner and Greenough, 1985). In animal models, exposure to such environment has shown to be beneficial for nervous system disorders, including brain injury (Johansson, 1996; Koopmans et al., 2006; Sale et al., 2009). Indeed, evidence suggests that the recovery of cognitive (e.g., learning and memory) and motor functions following experimental brain lesion is enhanced by this technique (Farrell et al., 2001; Hicks et al., 2002; Rönnbäck et al., 2005). Enriched environment following brain injury also has beneficial effects on the brain, such as decreasing lesion size or enhancing dendritic branching (Kolb and Gibb, 1991; Passineau et al., 2001; Nithianantharajah and Hannan, 2006).

## Sensory stimulation programs

Numerous studies investigated the impact of sensory stimulation programs on the recovery of patients with disorders of consciousness (DOC). However, when reviewing studies published from 1966 to 2002, Lombardi reported only three studies with adequate methodologies (Kater, 1989; Mitchell et al., 1990; Johnson et al., 1993), the other ones mostly being non-controlled designs or descriptive case reports. The results from this small number of studies could not confirm the efficacy of sensory stimulation programs (Lombardi et al., 2002). Indeed, besides an insufficient description of the program applied, the results were contradictory, the types and dosage of interventions but also the primary outcomes examined differed, making any study comparison difficult. Another bias was the role of spontaneous recovery. Indeed, these studies were mainly performed in the acute or subacute stage, a period during which spontaneous recovery has the highest probability to appear. Due to small sample sizes, none of these studies could ensure a dissociation between improvements attributed to the sensory stimulation treatment and improvements due to spontaneous recovery.

Since 2002, several studies investigated whether the improvements observed after treatment exceeded spontaneous recovery (Oh and Seo, 2003; Lotze et al., 2011; Di Stefano et al., 2012). Time-series designs were used since the treatment was compared to baselines (see Figure A1). Results showed more complex behavioral responses in the presence of treatment than in its absence, suggesting that sensory stimulation programs have truly an impact on the improvement of consciousness in patients recovering from coma. These studies nevertheless included a small number of patients (*n* < 15). Finally, only one study investigated the changes in brain activity related to treatment. Pape and colleagues examined the effects of a unimodal (auditory) stimulation program (Pape et al., 2015). They found better neurobehavioral performance in the treated group as compared with the control group. fMRI recordings performed before and after treatment demonstrated higher activation in the language network in the treated group as compared to the control group, suggesting an impact of the sensory stimulation program on the patients' brain recovery (see Figure A2). Such findings indicate that supplementing behavioral measures with neuroimaging may expand our understanding of the impact of sensory stimulation with such complex populations (see Table A1 in Appendix).

**Figure 1:**
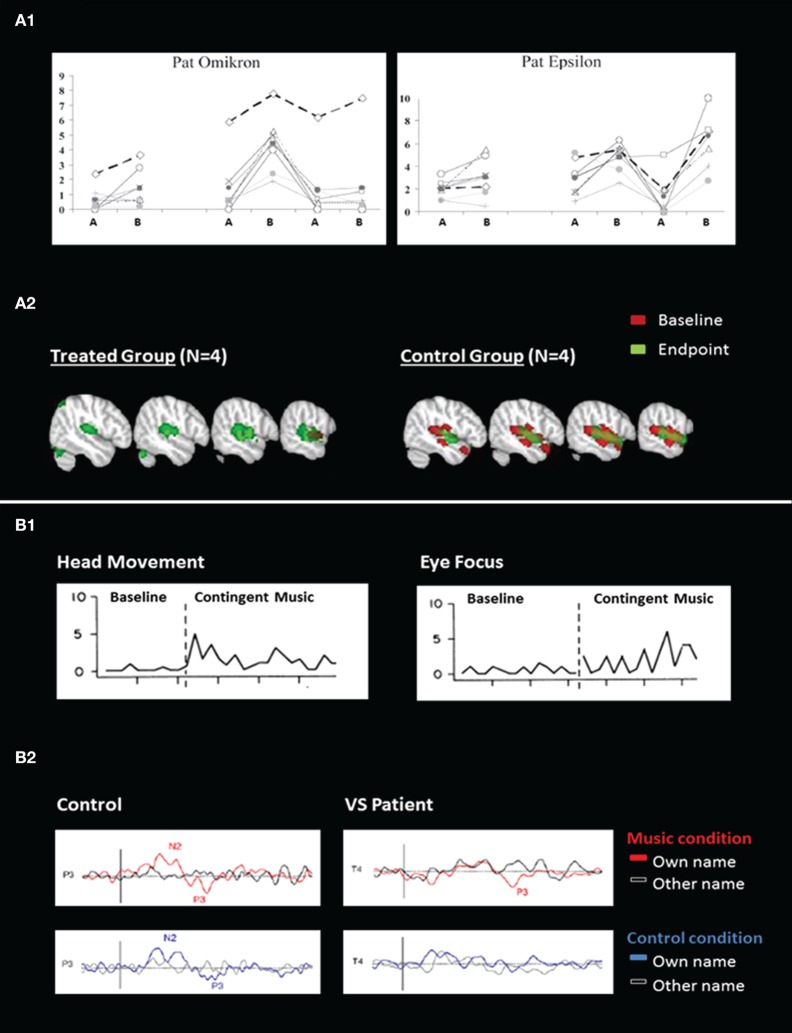
**Behavioral and neuroimaging responses to sensory stimulation (A) and music (B). (A1)** Illustrates averaged behavioral scores from blinded independent raters during multimodal sensory program in two patients. The x-axis describes time (ABABAB design where *A* = baseline and *B* = treatment) and the y-axis represents the rating scores (0 = no movement, 10 = voluntary movements; adapted from Lotze et al., 2011). **(A2)** Illustrates brain activation in response to unimodal sensory (auditory) stimulation, at the baseline and at the end of the study (adapted from Pape et al., 2015). **(B1)** Illustrates behavioral responses during baseline and music stimulation for head movements and eye focus in one patient (adapted from Boyle and Greer, 1983). **(B2)** Illustrates event-related potentials in response to the subject's own name and to other names in music and control conditions, in one control subject and in one patient in a vegetative state (VS) (adapted from Castro et al., 2015).

## A new potential option: music therapy

Music therapy interventions use live music that can be modified according to patient responsiveness “in the moment.” Musical parameters (e.g., tempo, rhythm) are manipulated according to changes in a patient's attention or arousal, incorporating salient content, such as the patient's name, in musical material. Salient auditory stimuli, such as family members' voices, increase the probability of observing brain and behavioral responses in DOC patients (Perrin et al., 2015). However, music listening may offer a superior auditory stimulus as it is believed to involve key areas supporting consciousness (Vanhaudenhuyse et al., 2010). Music's self-referential and autobiographical properties, in combination with stimulation of cognitive functions such as attention and mental imagery, may also act on these areas (Perrin et al., 2015). Previous studies with DOC populations showed that music enhanced arousal and attention when compared to white noise or disliked music (O'Kelly et al., 2013) or when compared to a control non-musical auditory stimulus (Castro et al., 2015), suggesting a potential impact of music therapy on consciousness recovery.

Research into music therapy with DOC has been limited due to the lack of behavioral measures that are sensitive to the complex needs of this population (Bradt et al., 2010; Magee and O'Kelly, 2015). For this reason, single subject designs and case reports prevail, reporting on behavioral and neurophysiological outcomes. A single case study assessed the effects of recorded music on a learned behavior through operant conditioning. Results demonstrated that music could be a motivating reward and could help when detecting signs of consciousness (Boyle and Greer, 1983; see Figure B1). Indeed, in another study patients had an increased cerebral response to their own name following a music condition in comparison to a control condition, suggesting that music can increase arousal and/or awareness (Castro et al., 2015; see Figure B2). Music stimulation activated superior temporal gyri in healthy adults (*n* = 21), minimally conscious patients (*n* = 2) and one patient in a vegetative state who recovered consciousness 4 months later, suggesting music's potential prognostic capacity in detecting conscious brain activity (Okumura et al., 2014).

Interventions using live music, typical in music therapy intervention, provide more promising data. Improvements in arousal and cognition during music therapy were noted in one study with 21 patients in DOC (O'Kelly et al., 2013). Personally salient live music resulted in significantly more eye blinks in VS patients when compared with baseline silence suggesting increased arousal. In the same study, *post-hoc* EEG amplitude increases were found for frontal midline theta and frontal alpha during the live presentation of personally salient music across both VS and MCS cohorts signaling greater cortical activity than responses to auditory stimuli of a non-salient nature (white noise and recordings of disliked music). Differential responses to live music vs. white noise indicated more intact cognitive processes suggestive of selective attention in the MCS cohort than the VS cohort where differentiation was less evident (O'Kelly et al., 2013). Another case report using standardized DOC behavioral measures compared responses in a DOC patient during neuropsychological evaluation with those measured during live music therapy interventions (Lichtensztejn et al., 2014). The results indicated that music therapy interventions at both baseline and post treatment elicited higher level responses involving behaviors demonstrating greater complexity, particularly within the auditory and language domains. These results are important in contributing to differential diagnosis in DOC patients. Music therapy interventions using live music may optimize the promising benefits that music as a stimulus in the auditory modality offers DOC patients (See Table A1 in Appendix).

## Limitations and perspectives

The beneficial effects of enriched environment on brain plasticity and cognitive functioning have been demonstrated by animal research. Its impact on human subjects is nevertheless much more challenging to show. The first difference is the control of the environment. Medication, changes in therapy, medical status or spontaneous recovery are among the variables the most difficult to control. Although, they are not impossible to account for, most studies examining sensory stimulation have been performed in an acute setting where those variables are in constant change. The inclusion of a chronic population would be a way to manage this bias as these patients are more stabilized. Indeed, changes in treatment or spontaneous recovery are not inexistent at a chronic stage but occur way less frequently.

The other weakness of these studies is the sample size. Most of them are case reports or descriptive case series which do not allow a generalization of the results. A longitudinal approach is useful when assessing the efficacy of treatment but such design require an important investment in time, making difficult for an isolated center to follow more than 30 cases simultaneously while finishing the study within a reasonable time-frame. A solution would be to develop an international initiative involving a significant amount of centers. This is not impossible since it has been done before for demonstrating the effect of Amantadine on the recovery of patients with severe brain injury (Giacino et al., 2012). This study was performed with the participation of 11 clinical sites and resulted in the recruitment of 184 patients which were followed during 6 weeks. The study used a randomized double-blind placebo-controlled design. Such sample size and such design represent a phase II clinical trial and allowed to establish the efficacy of the treatment.

The use of a controlled design may be more efficient when considering large samples since it requires a shorter follow-up. The use of a randomized (rather than matched) control group allows bias allocation to be minimized, balancing both known and unknown prognostic factors, in the assignment of treatments and is an optimal choice when dealing with such a heterogeneous population. Finally, one aspect that has been found useful in several preliminary studies (Castro et al., 2015; Pape et al., 2015) and should be considered in the future is the use of neuroimaging techniques (e.g., fMRI or electrophysiology; Giacino et al., 2014; Gosseries et al., 2014; Hannawi et al., 2015). Indeed, showing that treatment-related changes are observed using objective methods is essential to prove that sensory stimulation programs and music therapy are efficient in improving brain plasticity in patients with DOC.

## Conclusion

Initiating such a big project is challenging but is crucial since effective treatment options are limited. The combination of all these scientific findings will certainly help the clinicians to treat more efficiently patients with severe brain injury.

## Author contributions

All authors listed, have made substantial, direct and intellectual contribution to the work, and approved it for publication.

### Conflict of interest statement

The authors declare that the research was conducted in the absence of any commercial or financial relationships that could be construed as a potential conflict of interest.

## Appendix

**Table A1:** **Summary of previous studies investigating sensory stimulation program and music interventions**.

**Treatment**	**References**	**Sample size**	**Cause of injury**	**Time since injury**	**Level of consciousness**	**Design**	**Main findings after/during treatment**
Sensory stimulation program	Kater, 1989	30	TBI	2 weeks	Mix (GCS 3-14)	Non randomized controlled	Better outcome at 3 months post-injury
	Mitchell et al., 1990	24	TBI	4–12 days	Mix (GCS 4–6)	Non randomized controlled	Shorter duration of coma and increase in the GCS
	Johnson et al., 1993	14	TBI	<24 h	Mix (GCS =8)	Randomized controlled	No significant changes in the GCS, brainstem reflexes or physiological measurements
	Oh and Seo, 2003	5	TBI/NTBI	<3 months	Mix (GCS 3-7)	Time-series	Increase in the GCS
	Lotze et al., 2011	8	TBI/NTBI	16–126 months	Mix (VS/MCS)	Time-series	Improvements in behavioral responses (e.g., response to command)
	Di Stefano et al., 2012	12	TBI/NTBI	> One 1 month	Mix (VS/MCS)	Time-series	Greater range of behavioral responses based on the Wessex Head Injury Matrix
	Pape et al., 2015	15	TBI	Average of 70 days	Mix (VS/MCS)	Randomized controlled	Improvements in behavioral responses based on the Coma Near Coma Scale and in brain activity based on fMRI recording. Effect size: *d* = 1.88.
Music interventions	Boyle and Greer, 1983	3	TBI/NTBI	6–38 months	Mix (VS/MCS)	Operant conditioning	Changes in behavioral responses
	O'Kelly et al., 2013	21	TBI/NTBI	2–14 months	Mix (VS/MCS)	Multiple baseline within-subjects with randomized treatment order	Significant increases in blink rate to liked music in VS cohort; significant post hoc EEG amplitude in frontal midline theta and alpha for liked music in both VS and MCS cohorts
	Lichtensztejn et al., 2014	1	TBI	32 months	Mix (VS/MCS)	Case study	Changes in behavioral (including musical) responses
	Okumura et al., 2014	7	NTBI	12–72 months	Mix (VS/MCS)	Cross-sectional	Based on fMRI recording, activation of the superior temporal gyri to music in all MCS patients and in one of five VS patients who recovered consciousness 4 months later.
	Castro et al., 2015	13	TBI/NTBI	20 days to 3 years	Mix (VS/MCS)	Cross-sectional	Better cerebral (electrophysiological) responses in response to music and related to the outcome at 6 months

*TBI, Traumatic Brain Injury; NTBI, Non-Traumatic Brain Injury; GCS, Glasgow Coma Scale; VS, Vegetative State; MCS, Minimally Conscious State; fMRI, functional Magnetic Resonance Imaging; EEG, Electroencephalogram*.
